# Consumption of Sugar-Sweetened Beverages in Mississippi: Is There A Disparity? Behavioral Risk Factor Surveillance System, 2012

**DOI:** 10.3390/ijerph14030228

**Published:** 2017-02-24

**Authors:** Mina Qobadi, Marinelle Payton

**Affiliations:** Center of Excellence in Minority Health and Health Disparities, School of Health Sciences, Jackson State University, Jackson, MS 39213, USA; mina.qobadi@students.jsums.edu

**Keywords:** sugar-sweetened beverages (SSBs) intake, obesity, socio-demographic characteristics, behavioral factors, disparity

## Abstract

Although consumption of sugar-sweetened beverages (SSBs) is a key contributor to epidemic obesity and has dramatically increased over the past decade in the United States, little is known about its prevalence and associated factors. Data from the 2012 Behavior Risk Factor Surveillance System (BRFSS) were used to estimate the prevalence of SSB consumption and to explore the associations between socio-demographic characteristics, behavioral factors and SSB intake in Mississippi (*n* = 7220). Descriptive statistics, Chi-square tests and logistic regressions were conducted using SAS Proc Survey procedures, to account for the BRFSS′s multistage complex survey design and sample weights. Overall prevalence of self-reported daily SSB intake was 41.1%. Our findings showed that males (aOR = 1.4, 95% CI: 1.2–1.7, ref = female), blacks (aOR = 1.7, 95% CI: 1.4–2.1, ref = whites), adults aged 18–24 years (aOR = 5.0, 95% CI: 3.4–7.5, ref = 65 years or older), those with less than high school education (aOR = 1.9, 95% CI: 1.4–2.6, ref = college graduate), annual income <$25,000 (aOR = 1.3, 95% CI: 1.1–1.7, ref ≥ $50,000) and $25,000–49,999 (aOR = 1.3, 95% CI: 1.1–1.6, ref ≥ $50,000), those with no physical activity (OR = 1.3, 95% CI: 1.1–1.6, ref = physically active), daily smokers (aOR = 2.2, 95% CI: 1.7–2.7, ref = non-smokers), and those who reported eating at fast food or chain restaurants (aOR = 1.8, 95% CI: 1.2–2.5, ref = do not eat at fast food or chain restaurants) were more likely to consume SSBs, raising concerns about overweight and obesity in Mississippi.

## 1. Introduction

Obesity is a major health condition problem which has increased dramatically during the past years. In 1990, no state had a prevalence rate of obesity greater than 15%, whereas in 2006 only four states had rates of less than 40% [1]. By 2010, 36 states had obesity rates of 25% or higher, and 12 of those had a prevalence rate of obesity equal to or greater than 30%. Nationwide today, more than one-third (34.9%) of adults are obese [2]. Mississippi currently has the highest adult obesity rate in the nation (35%). According to the 2015 State of Obesity, the adult obesity rate in Mississippi has increased dramatically over the past years, from 15% in 1990 to 35.6% in 2015 and could reach up to 66.7% by 2030 [3].

While the etiology of obesity is complex, recent evidence suggests sugar-sweetened beverages (SSBs) including sucrose, high-fructose corn syrup, brown sugar, fruit-juice concentrates and other caloric sweeteners are the major sources of energy intake in the U.S. diet and contribute significantly to weight gain and obesity [4]. Evidence suggests that sugar-sweetened beverages are generally consumed quickly and do not trigger the same feeling of fullness that comes from solid food. Thus the calories we receive from SSBs tend to add to the total calories we consume in a day, rather than replacing solid ones [5]. The excess energy intake contributes to overweight and obesity because our body stores the extra calories as body fat. Prior research showed that SSB consumption increases weight gain in both children and adults. Malik et al. [6] in a systematic review of 32 cohort studies and randomized controlled trials (RCTs) found that one serving per day of SSB consumption is associated with a 0.06-unit increase in Body Mass Index (BMI) among children and an excessive weight gain of 0.12 to 0.22 kg among adults over one year Another study reported that higher intake of SSBs among children is significantly associated with 55% (95% CI: 32%–82%) increased risk of overweight or obesity [7]. In addition to obesity, research also suggests that SSB consumption is associated with diabetes mellitus [4,8], cardiovascular disease [9] and blood pressure [10].

Over the past three decades, SSB consumption has dramatically increased in the U.S. where one-half of the population is estimated to consume a sugar-containing beverage on any given day [4,11]. Based on the 2009–2010 the National Health and Nutrition Examination Survey (NHANES), U.S. adults consumed an estimated average of 151 kcal/day of SSB which is 7% of the total daily energy intake [12]. Although several studies have estimated SSB intake among a nationally representative sample of the U.S. population [12,13], only a few state-level studies have estimated SSB consumption [14]. Therefore, the primary aims of this study were to examine the prevalence of SSBs intake in Mississippi and to identify its associated factors.

## 2. Methods

### 2.1. Sample and Survey Administration

The 2012 Mississippi Behavioral Risk Factor Surveillance System (BRFSS) data was used in this cross-sectional study. BRFSS is an ongoing, state-based survey conducted by state health departments in collaboration with the Center for Disease Control and Prevention (CDC). It uses a multistage cluster sampling design based on random-digit dialing (landline and cell phone) to select a representative sample from each state′s non-institutionalized civilian residents aged 18 years or older. Data from each state are weighted to compensate for unequal probabilities of selection, adjust for non-response and non-coverage to match the sample to the population and to make representative population-based estimates.

BRFSS questions are designed to gather information from adults on their health condition and health-related behaviors. The questionnaire has three parts: (1) the core component; (2) optional modules; and (3) state-added questions. CDC requires states to ask all questions on the core questionnaire of each respondent. Optional modules are sets of questions on specific topics that states may choose to use on their questionnaires. Individual states may develop their own questions and add these questions to their questionnaires. These state-added questions are not edited or evaluated by CDC. Each year, the states and CDC agree on the content of the core component and optional modules.

In 2012, BRFSS included an optional module that Mississippi chose with two questions about SSB consumption. The questions allowed respondents to report on frequency of consumption of regular soda and fruit drinks, as the most commonly consumed types of SSBs [12]: “During the past 30 days, how often did you drink regular soda or pop that contains sugar? Do not include diet soda or diet pop.” and “During the past 30 days, how often did you drink sweetened fruit drinks, such as Kool-Aid, cranberry juice cocktail, and lemonade? Include fruit drinks you made at home and added sugar to”. For each question, respondents could report number of times per day, per week, or per month they consumed these beverages. A total of 7788 adults aged 18 years or older completed the SSB Optional Module.

### 2.2. Outcome Variable

The outcome measure was SSBs intake. The type of SSBs consumed was determined on the basis of survey respondents′ answers to two questions: (1) “During the past month, how often did you drink regular soda or pop that contains sugar? Do not include diet soda”; (2) “During the past month, how often did you drink sweetened fruit drinks, such as Kool-Aid (Kraft Foods), cranberry and lemonade? Include fruit drinks you made at home and added sugar to”; For each beverage type, adults reported the number of times per day, per week, or per month that they consumed each type of beverage. Weekly or monthly intake was converted to daily intake frequency by dividing weekly intake frequency by seven and monthly intake frequency by 30. To calculate frequency of total daily SSB intake, we summed the frequency of consumption of regular soda and fruit drinks. For bivariate analyses, responses were categorized as <1, or ≥1 time/day for total SSBs intake. The cut point of one time per day was chosen to define daily intake [15].

### 2.3. Explanatory Variables

Explanatory variables were socio-demographics (gender, age, marital and employment status, race, income and education level), healthy behaviors such as smoking, drinking, leisure-time physical activity and eating at fast-food or chain restaurants. For healthy behaviors, we used two leisure-time physical activity categories (physically active or physically inactive) and two eating behaviors categories (eat at fast food or chain restaurants or do not eat at fast food or chain restaurants). Smoking status was classified as daily smoker, some day smoker, former smoker, and nonsmoker and alcohol drinking was categorized as no drinking and any drinking (≥1 drink of any alcoholic beverage during the past month). For socio-demographic characteristics, we used four age groups (18–24, 25–44, 45–64, and ≥65 years), four levels of education (less than high school, high school, some college and college graduate), three employment categories (employed, unemployed and retired), and two groups for marital status (Married and Other). Taking into consideration that 98% of the population identified themselves as non-Hispanic black and non-Hispanic white, we used two groups for race (non-Hispanic white and non-Hispanic black) and excluded respondents of other races, leaving a final analytic sample of 7220 adults. Because a relatively large proportion (15%) of adults had “don’t know/refused/missing” responses for annual household income, we did not exclude these respondents from our analyses and we categorized annual household income as <$25,000, $25,000 to $49,999, ≥$50,000, or did not know/refused/missing. Missing data for other variables were verified and found to be less than 1%.

### 2.4. Statistical Analysis

We used χ^2^ tests to examine differences in SSBs consumption by socio-demographic characteristic and healthy behaviors. Simple logistic regression was used to examine unadjusted bivariate association of SSBs consumption, sociodemographic characteristics and healthy behaviors. Each predictor variable associated with SSBs consumption at *p* < 0.10 in the bivariate analyses was retained in the initial multiple logistic regression models [16]. Multiple logistic regression was conducted to estimate odds ratios for drinking SSB one or more times per day, adjusting for any potential confounders (socio-demographic variables and healthy behaviors). Correlations between all independent variables were examined prior to inclusion in the models, in order to avoid problems associated with multi-collinearity. The sample weight variable was applied to all analyses to provide valid estimates for the civilian non-institutionalized adult population. All statistical analyses were performed using SAS 9.4 (SAS Institute, Cary, NC, USA) adjusting for the complex sample design of the BRFSS. Statistical tests were determined to be significant for *p* values < 0.05.

## 3. Results

The participants’ characteristics are presented in Table 1. The respondents were predominantly female, non-Hispanic white, unmarried, employed, aged 45–64 years old, and had some college education and less than a $25,000 annual household income. The overall prevalence of self-reported daily SSB intake was 41.1%. Our findings (Table 1) indicated that the prevalence of daily SSB intake was significantly higher among males (45.3%), blacks (51.5%), unmarried adults (47.8%), those aged 18–24 years (63.0%), those with less than high school education (47.5%), those with less than a $25,000 annual income (48.2%), no physical activity (45.6%), and current smokers (58.9%). SSB intake was significantly lower among those who were retired (21.6%) and did not eat at fast-food restaurants (25.4%).

Unadjusted and adjusted ORs for the associations between risk factors and SSB intake are presented in Table 2. Model I (unadjusted model) showed that the likelihood of SSB intake was highest among males (OR = 1.4; 95% CI: 1.2–1.6, ref = female), blacks (OR = 2.0; 95% CI: 1.7–2.3, ref = whites), unmarried adults (OR = 1.8; 95% CI: 1.6–2.1, ref = married), aged 18–24 years (OR = 6.7, 95% CI: 5.0–8.9, ref = 64 years or older), those with less than high school education (OR = 2.3, 95% CI: 1.8–2.9, ref = college graduate), those with <$25,000 annual income (OR = 2.1, 95% CI: 1.7–2.5, ref = $50,000 or more), those who had no physical activity (OR = 1.3, 95% CI: 1.1–1.5, ref = physically active), daily smokers (OR = 2.3; 95% CI: 1.9–2.8; ref = non-smokers) and those who reported eating at fast-food restaurants (OR = 2.2; 95% CI: 1.6–2.8, ref = do not eat at fast-food restaurants). Those who were retired and former smokers were significantly associated with a reduced likelihood of SSB intake.

Table 2 also shows our final model adjusted for all cofounders. According to this model, males (aOR = 1.4, 95% CI: 1.2–1.7, ref = female), blacks (aOR = 1.7, 95% CI: 1.4–2.1, ref = whites), adults aged 18–24 years (aOR = 5.0, 95% CI: 3.4–7.5, ref = 65 years or older), those with less than high school education (aOR = 1.9, 95% CI: 1.4–2.6, ref = college graduate), those with no physical activity (aOR = 1.3, 95% CI: 1.1–1.6, ref = physically active), daily smokers (aOR = 2.2, 95% CI: 1.7–2.7, ref = non-smokers), and those who ate at fast-food or chain restaurants (aOR = 1.8, 95% CI: 1.2–2.5, ref = do not eat at fast-food or chain restaurants) had the greatest likelihood of SSB intake. Income had a marginally significant association with SSB intake; those with an annual household income of <$25,000 (aOR = 1.3, 95% CI: 1.1–1.7, ref ≥ $50,000) and $25,000–$49,999 (aOR = 1.3, 95% CI: 1.1–1.6, ref ≥ $50,000) were more likely to consume SSBs compare to those who had an annual income $50,000 or more. Marital and employment status had no association with SSB intake in the adjusted model.

## 4. Discussion

In 2012, about half of adults (41.1%) reported consuming SSBs at least once per day during the past month which is in line with the studies of Kit et al. [12] and Ogden et al. [13]. Consistent with prior studies, daily SSB consumption was most common among younger adults [13,17,18], men [13,17,18], non-Hispanic blacks [13,14,17], unmarried adults, those who had annual household income less than $25,000 [14] and those with less than a high school education. Furthermore, those who had no physical activity, smoked on a daily basis and ate at fast-food or chain restaurants had a higher rate of SSB intake [18].

The reasons for high SSB consumption in Mississippi could be due to differences in the food environment and beverage marketing factors. Research showed that there is a strong association between advertisement exposure and poor diet [19,20]. SSB companies, such as Coco-Cola and Pepsi, fast-food restaurants and grocery stores advertise SSB products more in the southern regions of the United States than companies do in other regions, which may encourage higher levels of SSB consumption among Mississippians [20]. In addition to advertisement exposure, availability and affordability have been shown to be key determinants of food choices and consumption [21,22]. Healthy foods increasingly cost more and fast-food restaurants have become increasingly available and cheaper, possibly increasing the likelihood of SSB consumption in Mississippi. Mississippi has the lowest annual household income in the nation, and low-income individuals may access SSBs and junk food more easily than other nutritious beverages and food because of differences in availability or prices. Furthermore, increased portion sizes and fast-food consumption are typical dietary behaviors of people in Mississippi which may increase the likelihood of SSB intake. Low literacy can be another reason for high SSB intake in Mississippi. Zoellner et al. [23], in a study conducted in the rural lower Mississippi Delta, found that adults with low health literacy consumed 230 kilocalories (kcal) per day of SSBs, whereas those who had adequate health literacy consumed 111 kcal per day of SSBs.

Similar to other studies, age and gender were strong predictors of consuming SSBs. Our findings indicated that younger adults were more likely to consume SSBs compared to older ones. One potential explanation for high SSB consumption among younger adults may be due to an emerging popularity of non-traditional SSBs among younger generations, their taste preference and greater exposure to SSB marketing. In addition, young people eat more outside the home and at fast-food or chain restaurants, increasing the possibility of SSB consumption. Gender differences in SSB consumption are not clear but research shows that men have higher intakes of all types of beverages than women [24] and they may be less sensitive and selective to the calories of what they drink. An additional key finding is the considerable variation in adult SSB consumption by race. SSB marketing targets black Americans more frequently relative to whites, which may result in their higher levels of consumption [25,26]. The SSB industry has been using a variety of marketing tools to target black consumers through advertising, sales promotions, scholarship programs, and sponsorship of events with black communities, as well as with the provision of employment opportunities [27]. This heavy marketing of SSB influences individuals’ preferences, purchase requests, and consumption. In addition to media and marketing, a lack of knowledge about calorie content could be another reason for racial disparity in SSB consumption. Prior research showed that African Americans had the lowest knowledge of calorie content of SSBs compared to whites [18]. Low health literacy and limited knowledge of SSB contribution to obesity can justify why people with less education are more likely to consume SSBs. Our findings also indicated that consumption of SSBs is associated with smoking and no physical activity. Although this association is unclear, smoking and less physical activity as markers of unhealthy behaviors can lead to lower-quality diets. Further research could help identify why these disparities exist and how they might be addressed.

The strength of this study is analyzing a population-based sample that is weighted to reflect the total population of Mississippi and can be extrapolated to states with similar demographic characteristics. However, the findings in this report are subject to several limitations. First, BRFSS is a cross-sectional questionnaire; therefore, causality and the direction of multivariate results cannot always be determined. Second, estimates of regular soda and fruit drink consumption were based on the respondents′ self-reports, which may be subject to recall and social desirability biases; therefore, estimates might be either underestimated or overestimated. Third, the consumption of only two types of SSBs (regular soda and fruit drinks) was examined in this study; other types of SSBs such as energy drinks and sweetened tea were not included. This study improves our understanding of adult SSB consumption by focusing on variations by sociodemographic characteristics. Additional research is needed to understand the underlying factors of increased SSB consumption among subgroups with higher intake.

## 5. Conclusions

Our findings indicated high prevalence of SSB consumption in Mississippi. Being male, black, younger, a smoker, having less education and no physical activity and eating at fast-food restaurants were associated with a greater likelihood of SSB consumption after adjusting for confounders. Reducing SSB consumption as part of a healthy lifestyle may reduce overweight and obesity and the risk for chronic diseases among Mississippi adults consequently. To reduce disparities in SSB intake, identifying variations in SSB consumption by subpopulation groups and the development of targeted interventions such as providing access to more healthful alternatives to SSBs is needed. Healthcare providers can support these efforts by developing educational materials to inform consumers about beverage options.

## Figures and Tables

**Table 1:** Respondents′ socio-demographic and behavioral characteristics at baseline and the prevalence of daily SSB intake by characteristics, The Behavioral Risk Factor Surveillance System 2012.

Characteristics	*N* (% ^a^)	Daily SSB Intake	*p*-Value ^b^
Total	7220 (100)	2227 (41.1)	
Gender			<0.0001
Male	2507 (46.8)	885 (45.3)	
Female	4713 (53.2)	1342 (37.4)	
Marital			<0.0001
Married	3520 (47.6)	974 (33.7)	
Unmarried	3700 (52.4)	1253 (47.8)	
Age (years)			<0.0001
18–24	313 (13.8)	198 (63.0)	
25–44	1357 (33.3)	651 (51.4)	
45–64	2916 (34.4)	865 (33.4)	
≥65	2634 (18.4)	513 (20.3)	
Education			<0.0001
<High School	1194 (19.3)	406 (47.5)	
High School	2352 (30.2)	794 (45.0)	
Some College	1873 (32.8)	595 (40.6)	
College	1801 (17.6)	432 (28.1)	
Race			<0.0001
White	4613 (60.6)	1148 (34.4)	
Black	2607 (39.4)	1079 (51.5)	
Income			<0.0001
<$25,000	2767 (44.2)	997 (48.2)	
$25,000–$49,999	1547 (25.2)	491 (41.5)	
≥$50,000	1813 (30.6)	442 (30.7)	
Employment			<0.0001
Employed	3605 (63.4)	1283 (44.3)	
Retired	2181 (15.6)	421 (21.6)	
Unemployed	1434 (20.7)	523 (46.2)	
Physical Activity			0.0007
Yes	4828 (69.7)	1392 (39.2)	
No	2392 (30.3)	835 (45.6)	
Smoking Status			<0.0001
Current Smoker	968 (17.9)	479 (58.9)	
Some Day Smoker	370 (6.0)	159 (52.1)	
Former Smoker	1808 (20.9)	426 (30.4)	
Non-smoker	4074 (55.1)	1163 (38.1)	
Drinking Alcohol			0.33
Yes	2208 (38.6)	718 (42.2)	
No	5012 (61.4)	1509 (40.4)	
Fast-food			<0.0001
Yes	6448 (92.7)	2069 (42.3)	
No	772 (7.3)	158 (25.4)	

^**a**^ Weighted Frequency. ^**b**^ *p*-values are Chi-squared.

**Table 2:** Association between SSB consumption, socio-demographic and behavioral characteristics, BRFSS 2012.

Characteristics	Unadjusted Model I ^a^	Adjusted Model II ^b^
Gender		
Male	1.4 (1.2–1.6)	1.4 (1.2–1.7)
Female	(ref)	(ref)
Marital Status		
Unmarried	1.8 (1.6–2.1)	1.0 (0.9–1.3)
Married	(ref)	(ref)
Race		
Black	2.0 (1.7–2.3)	1.7 (1.4–2.1)
White	(ref)	(ref)
Age(years)		
18–24	6.7 (5.0–8.9)	5.0 (3.4–7.5)
25–44	4.2 (3.5–5.0)	3.3 (2.5–4.4)
45–64	2.0 (1.7–2.3)	1.6 (1.2–2.0)
≥64	(ref)	(ref)
Education		
<High school	2.3 (1.8–2.9)	1.9 (1.4–2.6)
High school	2.1 (1.7–2.5)	1.5 (1.2–1.9)
Some college	1.8 (1.4–2.1)	1.3 (1.0–1.6)
College graduate	(ref)	(ref)
Income		
<$25,000	2.1 (1.7–2.5)	1.3 (1.1–1.7)
$25,000–$49,999	1.6 (1.3–2.0)	1.3 (1.1–1.6)
≥$50,000	(ref)	(ref)
Employment		
Retired	0.3 (0.3–0.4)	0.9 (0.7–1.1)
Unemployed	1.1 (0.9–1.2)	0.9 (0.7–1.2)
Employed	(ref)	(ref)
Physical Activity		
Inactive	1.3 (1.1–1.5)	1.3 (1.1–1.6)
Active	(ref)	(ref)
Smoking Status		
Daily Smoker	2.3 (1.9–2.8)	2.2 (1.7–2.7)
Some Day Smoker	1.8 (1.3–2.4)	1.6 (1.1–2.2)
Former Smoker	0.7 (0.6–0.8)	0.9 (0.8–1.2)
Non-smoker	(ref)	(ref)
Drinking Alcohol		
Yes	1.1 (0.9–1.3)	-
No	(ref)	-
Fast-food		
Yes	2.2 (1.6–2.8)	1.8 (1.2–2.5)
No	(ref)	(ref)

^**a**^ Unadjusted Model; ^**b**^ Adjusted for socio-demographic and behavioral variables excluding Drinking Status.
